# The new provisional WHO entity ‘*RUNX1* mutated AML' shows specific genetics but no prognostic influence of dysplasia

**DOI:** 10.1038/leu.2016.150

**Published:** 2016-06-17

**Authors:** T Haferlach, A Stengel, S Eckstein, K Perglerová, T Alpermann, W Kern, C Haferlach, M Meggendorfer

**Affiliations:** 1 MLL Munich Leukemia Laboratory, Munich, Germany; 2 MLL2, Praha, Czech Republic

*RUNX1* (runt-related transcription factor 1) is a myeloid transcription factor described as recurrently mutated in *de novo* acute myeloid leukemia (AML; ~10%), clustering in the intermediate-risk cytogenetic group and showing prognostic adverse impact on the overall survival and disease progression.^1, 2, 3^ In the World Health Organization (WHO) Classification of Tumors of Hematopoietic and Lymphoid Tissues,^4^ AML are classified in the categories ‘AML with recurrent genetic abnormalities', ‘AML with myelodysplasia-related changes (AML-MRC)', ‘therapy-related myeloid neoplasms' and ‘AML, not otherwise specified'. AML-MRC includes cases with a myelodysplasia-related cytogenetic abnormality, a previous myeloid malignancy or showing multilineage dysplasia (MLD). MLD positive (MLD^+^) morphology shows dysplastic features in ⩾50% of cells in ⩾2 hematopoietic lineages.^5^ In 2008 ‘AML with mutated *NPM1*' and ‘AML with mutated *CEBPA*' were introduced as provisional entities and reached in 2016 a status as own entities.^4, 5^ Therein, the presence of MLD alone will not classify a case as AML-MRC when one of these mutations is present. Ongoing discussions now focus on *RUNX1* mutations characterizing the new provisional entity ‘AML with mutated *RUNX1*'. However, classification of patients with MLD and *RUNX1* mutation into the AML-MRC category is questionable and needs to be discussed. We therefore comprehensively analyzed 152 *RUNX1*-mutated AML patients by cytogenetics and molecular genetics, and especially investigated the prognostic impact of MLD. *RUNX1*-mutated AML showed strong associations to trisomy 13 (13/152, 9%) and mutations within genes coding for the spliceosome (88/140, 63%), and for chromatin modifiers (86/140, 61%). However, MLD did not show prognostic impact in multivariate Cox regression analyses. This supports an approach to classify *RUNX1*-mutated AML also as a new provisional entity irrespective of dysplastic features.

In this study, we investigated 152 AML patients at diagnosis harboring a *RUNX1* mutation. The cohort comprised 49 female and 103 male, the median age was 67 years, ranging from 18 to 78 years. Ninety-nine percent of patients had a *de novo* AML and 1% a secondary AML. Therapy-related AML were excluded, as these are classified separately within the WHO. Forty out of 152 (26%) had allogeneic transplantation in the follow-up. All samples underwent May-Grünwald-Giemsa staining and cytochemistry. Dysplasia was assessed according to Goasguen *et al.*^6^ MLD was defined by ⩾50% dysplastic cells in ⩾2 lineages following the WHO guidelines.^4, 5^ In 132/152 patients all three lineages were evaluable, while in 20 cases only two hematopoietic lineages were evaluable. All patients were investigated by the standard chromosome banding analysis (cytogenetics) and a diagnostic molecular genetic approach following European Leukemia Network (ELN) guidelines.^7^ All patients had prognostically intermediate karyotypes according to Medical Research Council criteria (group 2).^8^ In addition, a next-generation sequencing-based mutational screening targeting 25 genes (Supplementary Table S2) was performed in 140/152 patients. All patients were intensively treated according to standard AML protocols.^9^ For further details and patients characteristics, see Supplementary Material. All patients gave written informed consent for research studies; the study design adhered to the tenets of the Declaration of Helsinki and was approved by the Institutional Review Board before its initiation.

Within the total cohort of 152 AML patients with *RUNX1* mutations, the majority were classified as M2 and M1 according to French-American-British (FAB) criteria^10^ (64/152, 42% and 45/152, 30%, respectively), followed by M0 (31/152, 20%), M4 (9/152, 6%) and M6 (3/152, 2%). This confirms earlier studies that described the immature and undifferentiated morphology of *RUNX1*-mutated AML, reflected by the high number of M0 subtypes, as well as a comparison to a matched control cohort without *RUNX1* mutation from our data base, showing only 2% of M0 cases (21/886, *P*<0.001; Supplementary Table S3).^11^ Addressing dysplasia revealed dysplastic granulopoiesis in 24% (37/152), erythropoiesis in 31% (47/152) and megakaryopoiesis in 55% (73/132) of patients. A total of 44 patients (33%) had no dysplastic features in any of the three cell lineages, 38 (29%) had unilineage dysplasia, 39 (30%) had bilineage dysplasia, whereas 11 cases (8%) had trilineage dysplasia (TLD). In four cases, a differentiation of bilineage dysplasia or TLD was not possible, as megakaryopoietic dysplasia was not evaluable. MLD was detected in 36% (54/152) of the analyzed bone marrow samples. These numbers are in line with a number of large AML studies, where MLD was found in 23–36% and TLD in 2–15% (Supplementary Table S4).

Chromosome banding analysis revealed cytogenetic aberrations in 59/152 (39%) patients. Thereof, 42 patients showed trisomies as sole abnormality, whereas only 17 showed other aberrations. In detail, 17 cases showed trisomy 8 (+8), 13 cases +13 and 4 patients each +11 and +14. Further four non-recurrent trisomies were observed. Only two cases had three aberrations, classifying for AML-MRC with complex karyotypes (⩾3 unrelated abnormalities). Although +8 is one of the most frequent recurrent cytogenetic aberrations in AML (10% of AML cases),^8^ +13 is a very rare event (~1% of AML);^12^ however, interestingly both show high incidences of *RUNX1* mutations.^1, 13^

The highest mutation frequency besides *RUNX1* mutations was observed for *ASXL1* (41%), followed by *SRSF2* (36%), *FLT3* (22% p.Asp835 and internal tandem duplication), *BCOR* (21%), *TET2* (18%), *IDH2* (17%) and *U2AF1* (16%). Mono-allelic *CEBPA* mutations were rarely detected (5%), double-mutated *CEBPA* was not identified, clearly differentiating these AML entities. *NPM1*-mutated cases (*n*=3) were excluded, as these cases qualify already for an own entity. Overall, 461 additional mutations were identified in 140 analyzed patients, resulting in a median number of three additional mutations (range: 0–6). Thus, in 98% of patients at least one additional mutation besides the *RUNX1* mutation was observed (Figure 1). Grouping the gene mutations to cellular pathways resulted in a high number of patients, harboring at least one mutation within the splicing machinery (63%), chromatin modification (61%), followed by DNA methylation (48%) and activated signaling (40%). The high incidence of mutations within the splicing machinery as well as chromatin modification is in line with very recently published data,^3^ as well as the high occurrence of trisomy 13 within this *RUNX1*-mutated cohort, where a high incidence of *SRSF2* and *ASXL1* mutations have also been described previously.^14^ However, these molecular genetic patterns occurred in our cohort also within cases with normal karyotype, indicating that rather *RUNX1* than +13 might be the trigger. In a very comprehensive study on 200 AML patients by whole-genome and whole-exome sequencing the respective pathways—splicing machinery and chromatin modification—were found to be mutated less frequently with 14% and 30%, respectively, indicating a specific genotype in *RUNX1*-mutated AML compared with overall AML.^15^ Assessing the classification according to Lindsley *et al.*^2^ would characterize these gene mutations as secondary type AML specific, whereas a *RUNX1* mutation itself is classified as *de novo*/pan AML alteration.

MLD^−^ patients showed a higher blast count than MLD^+^ cases (62 vs 46%, *P*<0.001), a higher incidence of +13 (12 vs 2% in MLD^+^, *P*=0.032), *IDH2* mutation (23 vs 8% in MLD^+^, *P*=0.035), but no *KIT* mutation (0 vs 8% in MLD^+^, *P*=0.016). All other clinical parameters, chromosomal alterations and additional gene mutations did not differ significantly between the MLD^−^ and MLD^+^ patients (Supplementary Table S1). Furthermore, there was no difference in *RUNX1* mutation localization and mutation type between MLD^+^ and MLD^−^ patients (Supplementary Figure S1).

In univariate analyses, the overall survival was adversely influenced by MLD^+^ (20 vs 31 months (mo), *P*=0.039), ⩾3 mutations in addition to *RUNX1* (20 vs 39 mo, *P*=0.003), mutations within the spliceosome (23 vs 43 mo, *P*=0.036), *DNMT3A* (20 vs 36 mo, *P*=0.032), *NRAS* (12 vs 31 mo, *P*=0.026) and *U2AF1* (21 vs 33 mo, *P*=0.039; Supplementary Figure S2). In multivariate Cox regression, only ⩾3 mutations retained the independent adverse prognostic influence (Table 1).

In conclusion, MLD^-^ and MLD^+^ *RUNX1*-mutated AML differ in some associations to genetic markers, such as +13 or *IDH2* mutation status without prognostic impact in multivariate analysis. However, in *RUNX1*-mutated AML, the overall pattern shows a specific landscape with high incidences of trisomies (such as +8 and +13), and mutations in the spliceosome and in chromatin modifiers, characterizing a unique secondary type AML phenotype.^2^ *RUNX1*-mutated AML shows shorter event-free survival,^1^ and we found ⩾3 mutations as independent prognostic marker influencing prognosis. However, the detection of MLD has no independent influence in multivariate analysis. We therefore strongly support the classification of *RUNX1*-mutated AML as a provisional entity in the new WHO classification, but without further consideration of dysplastic features such as MLD.

## Figures and Tables

**Figure 1:**
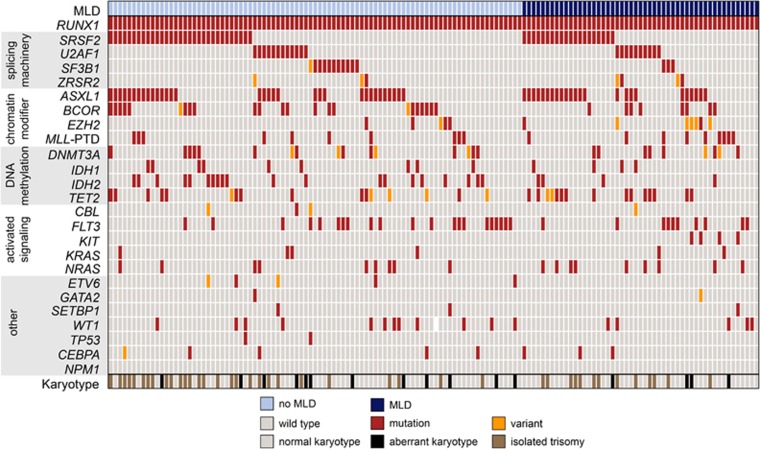
Molecular, cytogenetic and morphological characterization of AML patients with *RUNX1* mutation. Illustration of all 140 analyzed cases, each column represents one patient. All 25 analyzed genes, the occurrence of trisomies as sole aberration or other cytogenetic aberrations, as well as the presence of MLD (multilineage dysplasia) are given for each patient. *CEBPA* was single mutated in all mutated cases. Light blue: cases without MLD, dark blue: cases with MLD, light gray: wild-type gene and normal karyotype, red: mutation, orange: variant, brown: aberrant karyotype with isolated trisomy, black: other aberrant karyotype and white: no data available.

**Table 1:** Univariate and multivariate Cox regression analyses for the overall survival considering the covariates MLD and ⩾3 mutations, MLD and spliceosome mutations and MLD and mutations in the genes *DNMT3A*, *NRAS* and *U2AF1*

*Cox regression*								
	*Univariate*				*Multivariate*			
	P*-value*	*HR*	*95% CI*		P*-value*	*HR*	*95% CI*	
			*Lower*	*Upper*			*Lower*	*Upper*
*Overall survival*								
MLD	0.041	1.656	1.022	2.685	0.118	1.494	0.904	2.470
⩾3 mutations	0.003	2.176	1.297	3.649	0.005	2.108	1.255	3.539
MLD	0.041	1.656	1.022	2.685	0.099	1.528	0.923	2.529
Spliceosome mutations	0.038	1.763	1.031	3.015	0.050	1.714	1.001	2.935
MLD	0.041	1.656	1.022	2.685	0.066	1.634	0.969	2.755
*DNMT3A*	0.036	2.044	1.048	3.985	0.056	1.924	0.982	3.768
*NRAS*	0.031	2.389	1.085	5.258	0.058	2.246	0.972	5.190
*U2AF1*	0.042	1.898	1.023	3.521	0.203	1.541	0.792	3.001

Abbreviations: CI, confidence interval; HR, hazard ratio; MLD, multilineage dysplasia.
