# Supplementary material for: The new provisional WHO entity ‘RUNX1 mutated AML' shows specific genetics but no prognostic influence of dysplasia
Source: Leukemia. 2016 Jun 17;30(10):2109–12. doi: 10.1038/leu.2016.150 (PMC5056958; doi:10.1038/leu.2016.150)
Supplement: Supplementary Information [file leu2016150x1.pdf]

Supplemental Material

The new provisional WHO entity "*RUNX1* mutated AML" shows specific genetics without prognostic influence of dysplasia

Torsten Haferlach,[1] Anna Stengel,[1] Sandra Eckstein,[1] Karolína Perglerová,[2] Tamara Alpermann,[1] Wolfgang Kern,[1] Claudia Haferlach,[1] Manja Meggendorfer[1]

[1]MLL Munich Leukemia Laboratory, Munich, Germany

[2]MLL2, Praha, Czech Republic

1. Patients information

Bone marrow samples of all 152 patients were sent from different hematologic centers to the MLL Munich Leukemia Laboratory for diagnostics. Clinical characteristics as well as genetic information are given in Table S1 for the total *RUNX1* mutated AML cohort and differentiated by patients without and with MLD. Variants of unknown significance were excluded from statistical analyses and are therefore not listed in the table.

Table S1. Patients clinical characteristics, cytogenetics, and gene mutations.

Parameter (available cases n)	Patient numbers (% or ranges)		
	total cohort (n=152)	no MLD (n=98, 64%)	MLD (n=54, 36%)
Clinical characteristics (n=152)			
male/female (ratio)	103/49 (2.1)	64/34 (1.9)	39/15 (2.6)
median age (years)	67 (18-87)	67 (18-85)	66 (26-87)
median WBC count (x10^3/µl)	4.4 (0.4-211.8)	4.5 (0.6-196.0)	4.2 (0.4-211.8)
median platelet count (x10^3/µl)	80 (10-428)	78 (10-428)	90 (10-292)
median Hb level (g/dl)	8.9 (4.5-14.2)	8.9 (4.5-14.2)	8.9 (4.9-12.3)
bone marrow blasts (%)	59 (7-96)	63 (21-96)	40 (7-90)
Cytogenetics (n=152)			
normal karyotype	93 (61%)	57 (58%)	36 (67%)
aberrant karyotype	59 (39%)	41 (42%)	18 (33%)
trisomy 8	17 (11%)	11 (27%)	6 (33%)
trisomy 13	13 (9%)	12 (29%)	1 (5.5%)
trisomy 11	4 (3%)	2 (5%)	2 (11%)
trisomy 14	4 (3%)	1 (2%)	3 (17%)
other trisomies	4 (3%)	3 (7%)	1 (5.5%)
other aberrations	17 (11%)	12 (29%)	5 (28%)

Molecular mutations (n=140)			
ASXL1			
mutated	57 (41%)	33 (37%)	24 (47%)
wild type	83 (59%)	56 (63%)	27 (53%)
BCOR			
mutated	29 (21%)	21 (24%)	8 (16%)
wild type	109 (79%)	66 (76%)	43 (84%)
CBL			
mutated	1 (1%)	1 (1%)	0 (0%)
wild type	136 (99%)	86 (99%)	50 (100%)
CEBPA			
mutated (all single mutated)	7 (5%)	4 (5%)	3 (6%)
wild type	132 (95%)	84 (95%)	48 (94%)
DNMT3A			
mutated	19 (14%)	12 (14%)	7 (14%)
wild type	115 (86%)	73 (86%)	42 (86%)
ETV6			
mutated	3 (2%)	3 (3%)	0 (0%)
wild type	135 (98%)	84 (97%)	51 (100%)
EZH2			
mutated	6 (5%)	4 (5%)	2 (4%)
wild type	128 (95%)	84 (96%)	44 (96%)
FLT3 (p.Asp835 and internal tandem duplication, ITD)			
mutated	31 (22%)	21 (24%)	10 (20%)
wild type	109 (78%)	68 (76%)	41 (80%)
GATA2			
mutated	1 (1%)	1 (1%)	0 (0%)
wild type	138 (99%)	88 (99%)	50 (100%)
IDH1			
mutated	13 (9%)	9 (10%)	4 (8%)
wild type	127 (91%)	80 (90%)	47 (92%)
IDH2			
mutated	24 (17%)	20 (22%)	4 (8%)
wild type	116 (83%)	69 (78%)	47 (92%)
KIT			
mutated	4 (3%)	0 (0%)	4 (8%)
wild type	136 (97%)	89 (100%)	47 (92%)
KRAS			
mutated	6 (4%)	4 (5%)	2 (4%)
wild type	134 (96%)	85 (95%)	49 (96%)
MLL-PTD			
mutated	19 (14%)	10 (11%)	9 (18%)
wild type	121 (86%)	79 (89%)	42 (82%)
NPM1			
mutated	0 (0%)	0 (0%)	0 (0%)
wild type	140 (100%)	89 (100%)	51 (100%)
NRAS			
mutated	18 (13%)	9 (10%)	9 (18%)
wild type	122 (87%)	89 (90%)	42 (82%)
SETBP1			
mutated	3 (2%)	2 (2%)	1 (2%)
wild type	137 (98%)	87 (98%)	50 (98%)

SF3B1			
mutated	13 (9%)	10 (11%)	3 (6%)
wild type	126 (91%)	78 (89%)	48 (94%)
SRSF2			
mutated	51 (36%)	31 (35%)	20 (39%)
wild type	89 (64%)	58 (65%)	31 (61%)
TET2			
mutated	24 (18%)	11 (13%)	13 (27%)
wild type	109 (82%)	73 (87%)	36 (73%)
TP53			
mutated	2 (1%)	2 (2%)	0 (0%)
wild type	138 (99%)	87 (98%)	51 (100%)
U2AF1			
mutated	22 (16%)	12 (14%)	10 (20%)
wild type	118 (84%)	77 (86%)	41 (80%)
WT1			
mutated	17 (12%)	12 (14%)	5 (10%)
wild type	122 (88%)	76 (86%)	46 (90%)
ZRSR2			
mutated	3 (2%)	1 (1%)	2 (4%)
wild type	133 (98%)	86 (99%)	47 (96%)

2. *Cytomorphology*

All samples underwent May-Grünwald-Giemsa staining and cytochemistry (myeloperoxidase and nonspecific esterase). Dysplasia was assessed in granulopoiesis, erythropoiesis, and megakaryopoiesis according to Goasguen *et al.*[1] MLD was defined by ≥50% dysplastic cells in ≥2 lineages following the WHO guidelines.[2, 3] In 20 of 152 patients, only 2 hematopoietic lineages were evaluable, but patients could be defined as MLD^{+} in cases showing 2 dysplastic lineages or as MLD^{-} if 2 lineages were without dysplasia in 50% of cells. Therefore, all 152 patients were evaluable for MLD, while only 132 were evaluable for megakaryopoietic dysplasia.

3. *Next generation sequencing*

140/152 samples were investigated by a next generation sequencing (NGS) approach based on library preparation by the Access Array technology (Fluidigm, San Francisco, CA) and sequencing on the MiSeq Instrument (Illumina, San Diego, CA). The customized sequencing panel targeted 217 amplicons covering the hotspot or complete coding regions of the following 24 genes: *ASXL1*, *BCOR*, *CBL*, *CEBPA*, *DNMT3A*, *ETV6*, *EZH2*, *FLT3*, *GATA2*,

IDH1, *IDH2*, *KIT*, *KRAS*, *NPM1*, *NRAS*, *RUNX1*, *SETBP1*, *SF3B1*, *SRSF2*, *TET2*, *TP53*, *U2AF1*, *WT1*, *ZRSR2*. Gene mutations were annotated compared to the reference sequence based on the Ensembl Transcript ID (Ensembl release 74: Dec 2013). The transcript IDs as well as the targeted gene regions are given in Table S2.

Table S2: Targeted exons and transcript ID of the reference sequence for all by NGS investigated genes.

Gene	Sequenced exons	Transcript ID
ASXL1	E13	ENST00000375687
BCOR	complete coding region	ENST00000378444
CBL	E8, E9	ENST00000264033
CEBPA	complete coding region	ENST00000498907
DNMT3A	E7 - E23	ENST00000264709
ETV6	complete coding region	ENST00000396373
EZH2	complete coding region	ENST00000320356
FLT3	E20	ENST00000241453
GATA2	complete coding region	ENST00000341105
IDH1	E4	ENST00000345146
IDH2	E4	ENST00000330062
KIT	E17	ENST00000288135
KRAS	E2, E3	ENST00000256078
NPM1	E11	ENST00000296930
NRAS	E2, E3	ENST00000369535
RUNX1	complete coding region	ENST00000344691
SETBP1	E4	ENST00000282030
SF3B1	E11 - E16	ENST00000335508
SRSF2	E1	ENST00000392485
TET2	complete coding region	ENST00000380013
TP53	E4 - E11	ENST00000269305
U2AF1	E2, E6	ENST00000291552
WT1	E7, E9	ENST00000332351
ZRSR2	complete coding region	ENST00000307771

4. MLL-PTD and FLT3-ITD analyses

The partial tandem duplication (PTD) in the *MLL* gene was analyzed by quantitative PCR as described elsewhere.[4] The internal tandem duplication (ITD) in the *FLT3* gene was analyzed by fragment length analysis as described previously.[5]

5. Comparison of FAB subtypes to AML control cohort

For the comparison of the FAB subtype distribution within the *RUNX1* mutated cohort and a general AML cohort (MLL data set) we built a matched control cohort. This control cohort was selected from AML patients at diagnosis, intermediate cytogenetic MRC[6] class 2,

comparable therapy regime, and *RUNX1* wild type status (n=886). The control cohort comprised of 438 male and 448 female, the median age was 63 years (range: 18-88 years). The FAB classification[7] for both cohorts is given in Table S3.

Table S3. Comparison of FAB classification between *RUNX1* mutated AML and AML control cohort (MLL data set).

FAB classification	*RUNX1* mutated AML	AML control cohort*	*p*
M0	20% (31/152)	2% (21/886)	<0.001
M1	30 %(45/152)	36% (315/886)	n.s.
M2	42% (64/152)	33% (292/886)	0.033
M3	0% (0/152)	0% (1/886)	n.s.
M4	6% (9/152)	21% (187/886)	<0.001
M5	0% (0/152)	5%(47/886)	0.001
M6	2%(3/152)	2% (21/886)	n.s.
M7	0% (0/152)	0% (2/886)	n.s.

n.s.: not significant. * taken from MLL data set.

6. Comparison of MLD and TLD to other AML studies

All 152 patients were assessed for MLD. MLD was defined by ≥50% dysplastic cells in ≥2 lineages following the WHO guidelines. We compared our results to a number of large AML studies where MLD and TLD were addressed (TLD, trilineage dysplasia are also included in MLD). The percentage of patients with MLD and TLD are given in Table S4.

Table S4. Comparison of MLD and TLD to large published AML cohorts.

Study	Cohort	MLD	TLD
Haferlach *et al*[8]	AML	25%	15%
Miesner *et al*[9]	AML	36%	9%
Wandt *et al*[10]	AML	30%	9%
Bacher *et al*[11]	*CEBPA* mutated AML	26%	2%
Falini *et al*[12]	*NPM1* mutated AML	23%	5%
Present study	*RUNX1* mutated AML	36%	8%

MLD: multilineage dysplasia. TLD: trilineage dysplasia.

7. RUNX1 mutations in relation to MLD

The RUNX1 protein is encoded by six exons, 453 amino acids, and three main functional domains: the Runt domain, the transcription activation domain, and the transcription inhibition domain. The majority of patients harbored one *RUNX1* mutation (n=123; 81%),

while 29 (19%) showed two mutations within the *RUNX1* gene. Mutations in the *RUNX1* gene are distributed all over the coding sequence. For comparison of mutation type and localization in no MLD and MLD cases, the mutations are plotted separately (Figure S1). There are no differences detectable between these two groups.

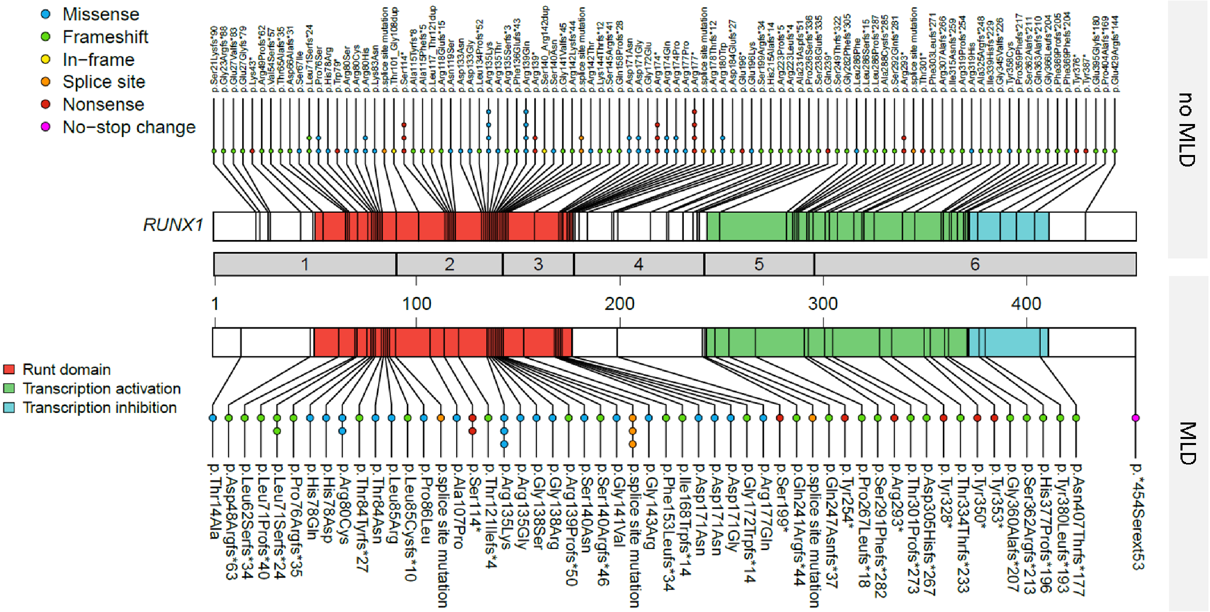

Figure S1. Mutation types and location within the *RUNX1* gene. Every single mutation is plotted. Upper part demonstrates no MLD, lower part MLD. MLD: multilineage dysplasia.

8. Overall survival analyses

We addressed the prognostic influences of all analyzed clinical and genetic markers within the *RUNX1* mutated AML cohort by Kaplan-Meier analyses. The median follow up of the total cohort was 25.5 months. MLD, additional mutations ≥3, mutations in at least one of the spliceosomal genes, *DNMT3A*, *NRAS*, and *U2AF1* turned out to adversely affect overall survival. However, in multivariate Cox regression analysis, only ≥3 mutations retained the independent adverse prognostic influence. The Kaplan-Meier plots are shown in Figure S2.

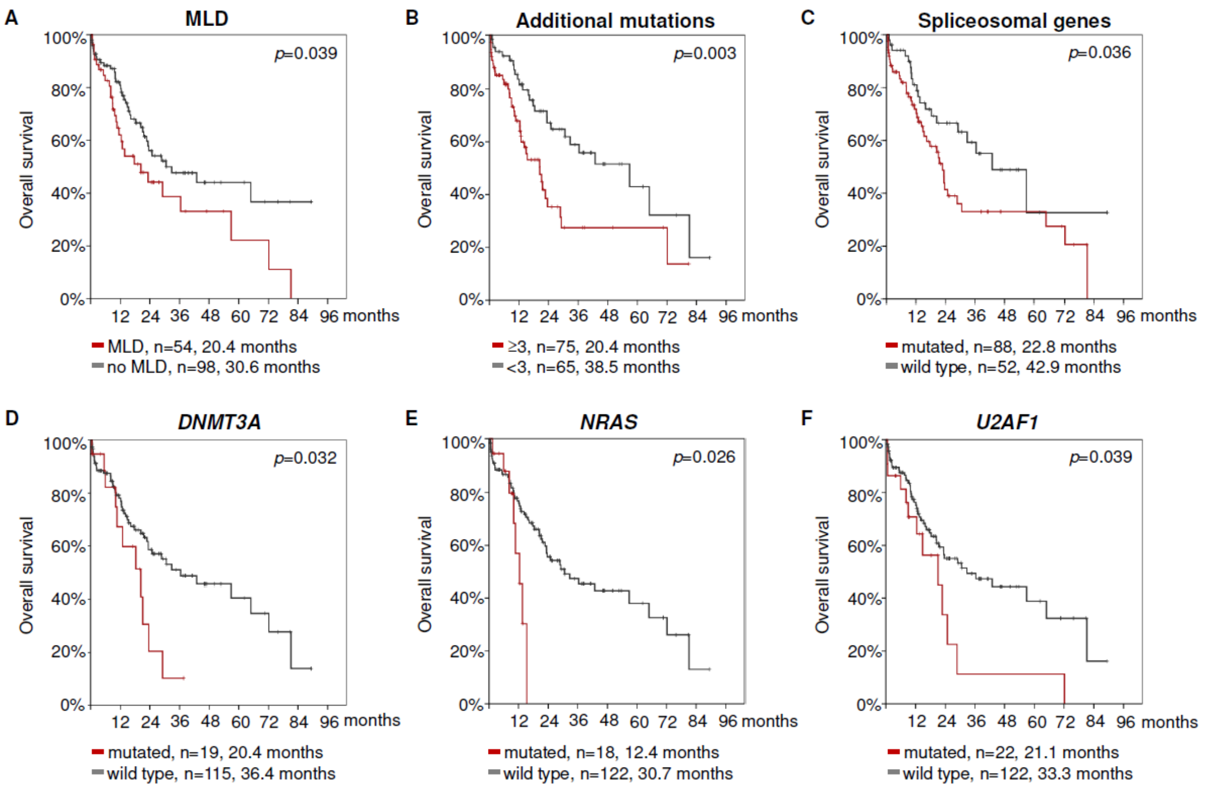

Figure S2. Overall survival analyses of different clinical markers. The case numbers and median overall survival are given beneath the Kaplan-Meier plots, respectively. Significant *p*-values are given. A) MLD vs no MLD. B) Additional gene mutations ≥3 vs <3. C) Mutation in at least one spliceosomal gene vs no mutation in any spliceosomal gene. D) *DNMT3A* mutated vs wild type. E) *NRAS* mutated vs wild type. F) *U2AF1* mutated vs wild type. MLD: multilineage dysplasia.

9. References

1 Goasguen JE, Matsuo T, Cox C, Bennett JM. Evaluation of the dysmyelopoiesis in 336 patients with de novo acute myeloid leukemia: Major importance of dysgranulopoiesis for remission and survival. *Leukemia* 1992; **6**: 520-525.

2 Swerdlow SH, Campo E, Harris NL, Jaffe ES, Pileri SA, Stein H, et al. WHO Classification of Tumours of Haematopoietic and Lymphoid Tissues. 4th ed. Lyon: International Agency for Research on Cancer (IARC); 2008.

3 Arber DA, Orazi A, Hasserjian R, Thiele J, Borowitz MJ, Le Beau MM, et al. The 2016 revision to the World Health Organization (WHO) classification of myeloid neoplasms and acute leukemia. *Blood* 2016.

4 Weisser M, Kern W, Schoch C, Hiddemann W, Haferlach T, Schnittger S. Risk assessment by monitoring expression levels of partial tandem duplications in the MLL gene in acute myeloid leukemia during therapy. *Haematologica* 2005; **90**: 881-889.

5 Schnittger S, Schoch C, Dugas M, Kern W, Staib P, Wuchter C, et al. Analysis of FLT3 length mutations in 1003 patients with acute myeloid leukemia: correlation to cytogenetics, FAB subtype, and prognosis in the AMLCG study and usefulness as a marker for the detection of minimal residual disease. *Blood* 2002; **100**: 59-66.

6 Grimwade D, Hills RK, Moorman AV, Walker H, Chatters S, Goldstone AH, et al. Refinement of cytogenetic classification in acute myeloid leukemia: determination of prognostic significance of rare recurring chromosomal abnormalities among 5876 younger adult patients treated in the United Kingdom Medical Research Council trials. *Blood* 2010; **116**: 354-365.

7 Bennett JM, Catovsky D, Daniel MT, Flandrin G, Galton DA, Gralnick HR, et al. Proposed revised criteria for the classification of acute myeloid leukemia. A report of the French-American-British Cooperative Group. *Ann Intern Med* 1985; **103**: 620-625.

8 Haferlach T, Schoch C, Loffler H, Gassmann W, Kern W, Schnittger S, et al. Morphologic dysplasia in de novo acute myeloid leukemia (AML) is related to unfavorable cytogenetics but has no independent prognostic relevance under the conditions of intensive induction therapy: results of a multiparameter analysis from the German AML Cooperative Group studies. *J Clin Oncol* 2003; **21**: 256-265.

9 Miesner M, Haferlach C, Bacher U, Weiss T, Macijewski K, Kohlmann A, et al. Multilineage dysplasia (MLD) in acute myeloid leukemia (AML) correlates with MDS-related cytogenetic abnormalities and a prior history of MDS or MDS/MPN but has no independent prognostic relevance: a comparison of 408 cases classified as "AML not otherwise specified" (AML-NOS) or "AML with myelodysplasia-related changes" (AML-MRC). *Blood* 2010; **116**: 2742-2751.

10 Wandt H, Schakel U, Kroschinsky F, Prange-Krex G, Mohr B, Thiede C, et al. MLD according to the WHO classification in AML has no correlation with age and no independent prognostic relevance as analyzed in 1766 patients. *Blood* 2008; **111**: 1855-1861.

11 Bacher U, Schnittger S, Macijewski K, Grossmann V, Kohlmann A, Alpermann T, et al. Multilineage dysplasia does not influence prognosis in CEBPA-mutated AML, supporting the WHO proposal to classify these patients as a unique entity. *Blood* 2012; **119**: 4719-4722.

12 Falini B, Macijewski K, Weiss T, Bacher U, Schnittger S, Kern W, et al. Multilineage dysplasia has no impact on biologic, clinicopathologic, and prognostic features of AML with mutated nucleophosmin (NPM1). *Blood* 2010; **115**: 3776-3786.
